# Construction of a Cosmid-Based Ultraefficient Genomic Library System for Filamentous Fungi of the Genus *Aspergillus*

**DOI:** 10.3390/jof10030188

**Published:** 2024-02-29

**Authors:** Chihiro Kadooka, Takuji Oka

**Affiliations:** Department of Biotechnology and Life Sciences, Faculty of Biotechnology and Life Sciences, Sojo University, Ikeda 4-22-1, Nishi-ku, Kumamoto 860-0082, Japan

**Keywords:** cosmid vector, genomic library, *Aspergillus*, classical genetics

## Abstract

Filamentous fungi of the genus *Aspergillus* include producers of industrially important organic acids, enzymes, and secondary metabolites, as well as pathogens of many plants and animals. Novel genes in the *Aspergillus* genome are potentially crucial for the fermentation and drug industries (e.g., agrochemicals and antifungal drugs). A research approach based on classical genetics is effective for identifying functionally unknown genes. During analyses based on classical genetics, mutations must be identified easily and quickly. Herein, we report the development of a cosmid-based plasmid pTOCK1 and the use of a genomic library of *Aspergillus nidulans* constructed using pTOCK1. The cosmid-based genomic library was used for convenient auxotrophic mutants (*pyroA* and *pabaB*), as well as mutants with abnormal colony morphology (*gfsA*) and yellow conidia (*yA*), to obtain library clones complementary to these phenotypes. The complementary strain could be obtained through a single transformation, and the cosmid could be rescued. Thus, our cosmid library system can be used to identify the causative gene in a mutant strain.

## 1. Introduction

Filamentous fungi of the phylum Ascomycetes, including *Aspergillus*, are among the most diverse and species-rich groups of organisms on Earth [1,2]. Filamentous fungi have varying characteristics. They can (1) cause serious diseases in plants and animals, (2) be used to ferment foods, and (3) produce organic acids, enzymes, and secondary metabolites, including antibiotics [3,4]. Therefore, identifying unique genes that determine the characteristics of filamentous fungi is vital for developing sustainable societies.

With the release of genome information on *Aspergillus nidulans*, *Aspergillus oryzae*, and *Aspergillus fumigatus* in the early 2000s, genome information is now available for several *Aspergillus* spp. [5,6,7]. The development of hosts with mutant genes encoding Ku70/Ku80 heterodimers and DNA ligase IV—which are involved in nonhomologous end-joining—has enabled the knockout of genes with high efficiency [8,9,10,11]. Advances in genome editing technologies, such as the CRISPR/Cas9 system, have eased the use of gene disruption in industrial and clinical strains, as well as model strains [12]. Therefore, reverse genetics has revealed the function of many genes in pathogenic and industrially used filamentous fungi. However, although reverse genetics is a powerful tool, it is unsuitable for analyzing functionally unknown genes without functional information of homologs.

Filamentous fungi were once a leading research tool in genetics, exemplified by the one gene–one enzyme hypothesis in the red bread mold, *Neurospora crassa* [13,14,15]. *A. nidulans*, the model *Aspergillus* species, was isolated by Pontecorvo at the University of Glasgow as a fungus with sexual development, thereby, as a species for performing genetics experiments [16]. Therefore, experimental procedures for genetic analysis are more highly developed for *A. nidulans* than other filamentous fungi [17]. However, the development of genetic research for *A. nidulans* has been delayed compared with *Saccharomyces cerevisiae* and *Schizosaccharomyces pombe* owing to the transformation difficulty and lack of naturally occurring self-replicating plasmids. Several multicopy suppressors have been identified in species such as *Saccharomyces cerevisiae* using a combination of high-copy plasmids and a strong inducible promoter [18]. Groups of genes involved in filamentous fungus-specific differentiation and asexual spore formation, such as BrlA/AbaA/WetA pathway genes, StuA/MedA, FluG, and FlbA–E, have been discovered by classical genetics in *A. nidulans* [19]. However, many unidentified gene functions potentially encode proteins of unknown function in the filamentous fungus genome, which lack homologs in yeast. These genes possibly confer filamentous characteristics to filamentous fungi but are difficult to identify using reverse genetics. Therefore, phenotype-based classical genetics is required to annotate and characterize gene function in filamentous fungi.

Highly efficient genomic libraries are essential for rapid classical genetics; ones containing the autonomous replication origin *AMA1* were distributed by the Fungal Genetics Stock Center. However, this resource is now unavailable because reamplifying this genomic library by introduction into *Escherichia coli* is challenging because of the large size of the plasmids. Punt et al. at Leiden University have constructed a cosmid-based *AMA1* genomic library for *Aspergillus niger* to identify the genes responsible for the mutant strains [20]. However, this cosmid library is not readily available because it is maintained in the laboratory of Punt. Cosmid libraries have longer insert lengths than plasmid libraries and thus can achieve higher genome coverage. They can be easily amplified by culturing *E. coli*, and a small amount of the library solution can be used to transform *E. coli* efficiently with lambda phage. Indeed, *yA*, which encodes conidial laccase, was identified in a cosmid clone [21]. In addition, the cosmid library was used to identify *laeA*, which encodes a master regulator of secondary metabolic biosynthesis with the greatest impact on filamentous fungi recently [22]. Clones complementing the phenotypes of *A. nidulans* mutants were identified using the cosmid library of *A. fumigatus* [23]. The cosmid library has high potential because although the cosmid vectors do not contain an *AMA1* insertion, genes can be identified. We hypothesized that an ultraefficient genomic library for *Aspergillus* spp. could be produced by constructing a cosmid vector containing *AMA1*, which is known to significantly increase transformation efficiency [24].

Herein, we constructed a cosmid-based *AMA1* genomic library using *A. nidulans* genomic DNA and tested its efficiency. Auxotrophic complementation experiments of *pyroA* (pyridoxine) and *pabaB* (4-hydroxybenzoate) and complementation experiments with galactofuranosyltransferase (*gfsA*) and *yA* mutants yielded the desired strains after a single transformation. A small amount of genomic DNA was extracted from the colonies of each strain, and the target cosmid DNA was recovered quickly and easily by in vitro packaging. In addition, small amounts of cosmid DNA could be reintroduced into *E. coli* to reamplify the genomic library with high efficiency. Thus, the construction of a highly efficient cosmid library suitable for this purpose is expected to advance classical genetics-based studies of filamentous fungi.

## 2. Materials and Methods

### 2.1. Strains and Medium

The *A. nidulans* strains used in this study are listed in Table 1. The strains were cultured on *Aspergillus* minimal medium (MM; 1% glucose [wt/vol], 0.6% NaNO_3_ [wt/vol], 0.052% KCl [wt/vol], 0.052% MgSO_4_·7H_2_O [wt/vol], 0.152% KH_2_PO_4_ [wt/vol], and Hutner’s trace elements [pH 6.5]). For culturing AKU89 [25], 1 g/L arginine and 5 μg/L biotin were added to MM. For culturing *A. nidulans* AKU89PPAP, 1.22 g/L uracil, 1.21 g/L uridine, 5 μg/L pyridoxine HCl, and 5 μg/L 4-aminobenzoic acid were added to MM.

*E. coli* DH5 alpha cells were used for vector construction and maintenance. The *A. nidulans* cosmid library pTOCK1-gAKU89 was constructed and propagated in *E. coli* ED8767. 

### 2.2. Construction of the Shuttle Cosmid Vector pTOCK1

**Figure 1:**
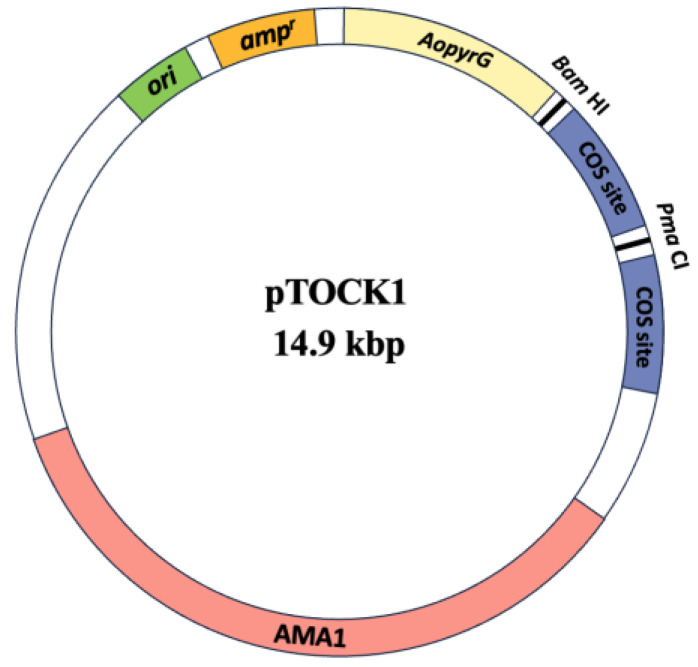
Cosmid vector map of pTOCK1. The DDBJ/ENA/GenBank accession number is LC795782. The addgene catalog number is #215566.

Shuttle vectors for *Aspergillus* spp. and *E. coli* were constructed using SuperCos1 Cosmid Vector Kit (Agilent Technologies, Santa Clara, CA, USA). To replace the *Xba*I site of SuperCos1 with the *Pma*CI site, SuperCos1 was amplified by inverse PCR using the SuperCos1 template and the SuperCos1-PmaCI-F2–SuperCos1-PmaCI-R2 primer pair (Table 2). Amplification products were *Dpn*I treated, phosphorylated with T4 Polynucleotide Kinase (Nippon Gene Co., Ltd., Tokyo, Japan), and self-ligated using DNA ligase (Nippon Gene) to yield SuperCos1-PmaCI. Orotidine-5′-phosphate decarboxylase gene (*pyrG*) from *A. oryzae* was amplified by PCR using *A. oryzae* RIB40 genomic DNA as a template, and the AoPyrG-Scos1-infusion(AatII)-F–AoPyrG-Scos1-infusion(AatII)-R primer pair (Table 2). The amplified DNA was cloned into the *Aat*II site of SuperCos1-PmaCI using In-Fusion HD Cloning Kit (TaKaRa Bio, Kusatsu, Japan) to yield SuperCos1-AopyrG. A *Hin*dIII fragment of pHELP1 [24] containing *AMA1* was ligated with *Hin*dIII-digested SuperCos1-AopyrG to yield pTOCK1 (Figure 1). The pTOCK1 sequence was obtained using Oxford Nanopore sequencing technology with a 2174-fold coverage. Sequence assembly was performed using Plasmid Verification Pipeline v1.0 (Eurofins Genomics KK, Tokyo, Japan). The plasmid sequence has been deposited in DDBJ/ENA/GenBank under the accession number LC795782. The pTOCK1 plasmid is available via the Addgene plasmid repository (catalog number #215566).

### 2.3. Construction of the A. nidulans Cosmid Library pTOCK1-gAKU89

*A. nidulans* AKU89 [25] was incubated in 100 mL of liquid MM, supplemented with arginine and biotin, at 37 °C with shaking (127 rpm) for 24 h and harvested by filtration. Mycelia were powdered using a mortar and pestle in liquid nitrogen. Wet powdered mycelia (1 g) were suspended in 15 mL of extraction buffer (200 mM Tris-HCl [pH8.5], 250 mM NaCl, 25 mM EDTA, and 0.5% SDS [wt/vol]), mixed gently by tipping up and down, mixed with equal volumes of phenol:chloroform (1:1), and centrifuged at 10,000× *g* and 4 °C for 15 min. The supernatant was collected and dialyzed in distilled water overnight using a dialysis membrane (Nihon Medical Science Inc., Osaka, Japan). The genomic DNA solution was concentrated using a centrifugal concentrator. In total, 1 mg of RNAase-treated genomic DNA was partially digested using *Sau*3AI. Agarose gel electrophoresis confirmed the digestion of genomic DNA into 10–40 kbp long fragments.

The cosmid vector pTOCK1 was digested with *Pma*CI, treated with calf-intestinal alkaline phosphatase (Nippon Gene), and digested with *Bam*HI. pTOCK1 and *Sau*3AI-digested genomic DNA were ligated using the Ligation-Convenience Kit (Nippon Gene). The ligation mix was packaged in vitro using LAMBDA INN in vitro Packaging Kit (Nippon Gene) and incorporated into the reconstituted lambda phage. The reconstituted lambda phage was mixed with *E. coli* ED8767 for infection. The mixture was placed on an LB medium containing 50 μg/mL ampicillin. All emerged colonies (approximately 54,000 colonies) were gathered, aliquoted, mixed with 50% glycerol, and stored at −80 °C. A small amount of stock solution was incubated in 1000 mL of liquid LB medium containing 50 μg/mL ampicillin, and plasmid extraction was performed using NucleoBond Xtra Maxi (TaKaRa Bio). The resulting 3 mg of DNA was suspended in 1 mL of sterile water and used to transform *A. nidulans*.

### 2.4. Construction of AKU89P

*pyrG* was disrupted in *A. nidulans* AKU89 through ORF deletion. A gene replacement cassette encompassing the homologous arms at the 5′ and 3′ ends of *pyrG* was amplified by recombinant PCR using *A. nidulans* A26 genomic DNA as a template, and the pyrG-del-1–pyrG-del-2 and pyrG-del-3–pyrG-del-4 primer pairs, respectively (Table 2). DNA amplified with the pyrG-del-1 and pyrG-del-4 primers was transformed into *A. nidulans* AKU89, yielding AKU89P. Transformants were cultured on nonselective regeneration MM medium supplemented with uracil and uridine and transferred to selective MM agar plates supplemented with 0.05% 5-fluoroorotic acid monohydrate, uracil, and uridine [27]. Deletion of *pyrG* into each locus was confirmed by PCR using the primer pair pyrG-FC–pyrG-RC.

### 2.5. Construction of AKU89PPAP

AKU89P and A426 were crossed (Table 1) [17]. Briefly, AKU89P and A426 conidia were inoculated adjacent to each other on MM agar medium, supplemented with arginine, biotin, uracil/uridine, pyridoxine, and 4-aminobenzoic acid, and incubated for 2 weeks at 37 °C in the dark. Some cleistothecia were picked up, hyphal and hüle cells were removed on 2% water agar, and ascospores were extracted by crushing in 100 µL sterile water. Small suspensions were inoculated onto MM agar medium supplemented with suitable nutrients, and those in which green and yellow colonies appeared were selected. Several auxotrophic strains of arginine, biotin, uracil/uridine, pyridoxine, and 4-aminobenzoic acid were selected and designated AKU89PPAP.

### 2.6. Construction of the gfsA Mutant Strain

*gfsA* was mutated in *A. nidulans* AKU89P by *argB* insertion. A gene replacement cassette was amplified by PCR using Δ*gfsA* (AN8677) genomic DNA [26] as a template and AN8677-7 and AN8677-8 as primers (Table 2). The amplified DNA was used to transform *A. nidulans* AKU89P, yielding Δ*gfsA,* Δ*pyrG*. The MM agar plates were used to select transformants. The introduction of *gfsA* into each locus was confirmed by PCR using the primer pair AN8677-FC–AN8677-RC.

### 2.7. Construction of pHSG396-nat1

*Streptomyces noursei* nourseothricin *N*-acetyl transferase (*nat1*) was amplified by PCR using pD-NAT1 as a template and the nat1-F–nat1-R primer pair [28]. pHSG396-hygB [29], lacking *hph* ORF, was amplified by inverse PCR using pHSG396-hygB as a template and the AnGpdA-P-TrpC-T-F(nat1)–AnGpdA-P-TrpC-T-R(nat1) primer pair (Table 2). The amplified fragments were assembled using In-Fusion HD Cloning Kit to yield pHSG396-nat1.

### 2.8. Construction of AKU89PY

*yA* was disrupted in *A. nidulans* AKU89P by *nat1* insertion. A gene replacement cassette, encompassing homologous arms of *yA* at 5′ and 3′ ends, was amplified by recombinant PCR using *A. nidulans* A26 genomic DNA as a template and the primer pairs yA-1–yA-2 and yA-3–yA-4, respectively. The *nat1* marker was amplified by recombinant PCR using pHSG396-nat1 as a template and the pHSG396-F–pHSG396-R primer pair [29]. The fragment amplified with yA-1, and yA-4 was used to transform *A. nidulans* AKU89P to yield AKU89PY strains. The MM agar plates supplemented with 2 mg/mL nourseothricin sulfate (clonNAT) (Jena Bioscience GmbH, Jena, Germany) were used to select transformants.

## 3. Results

### 3.1. Construction of the A. nidulans Genomic Library pTOCK1-gAKU89

To demonstrate the efficiency of the cosmid library, pTOCK1-gAKU89 was constructed by inserting a genomic DNA fragment of *A. nidulans* AKU89 into pTOCK1. Partially digested genomic DNA fragments (10–40 kbp long) were prepared by diluting *Sau*3AI with 1 mg of genomic DNA. Subsequently, 1 μg of pTOCK1 digested with *Pma*CI and *Bam*HI was ligated with 2.5 μg of *Sau*3AI-digested genomic DNA. Reconstituted lambda phage was constructed and infected with in vitro packaging of the ligation solution and *E. coli* ED8767, respectively. The length of DNA incorporated into the lambda phage head by in vitro packaging is 36–52 kbp. Because the base length of pTOCK1 was 14.9 kbp (Figure 1), the theoretical length of the insert DNA was 21.1–37.1 kbp. Approximately 54,000 clones were obtained, and pTOCK-gAKU89 was prepared by plasmid extraction. It should be noted that when several clones of pTOCK1-gAKU89 were sequenced by Oxford Nanopore sequencing technology, there were clones in which multiple fragments of different chromosomal origin were inserted. Transformation efficiency of pTOCK1-gAKU89 in *Aspergillus* spp. was high, depending on the autonomous replication sequence of *AMA1* [30], ranging from 2.0 to 4.0 × 10^5^ cells/µg DNA. 

### 3.2. Complementation Analysis of Pyridoxine- and 4-Aminobenzoate-Auxotrophic Strains Using pTOCK1-gAKU89

**Figure 2:**
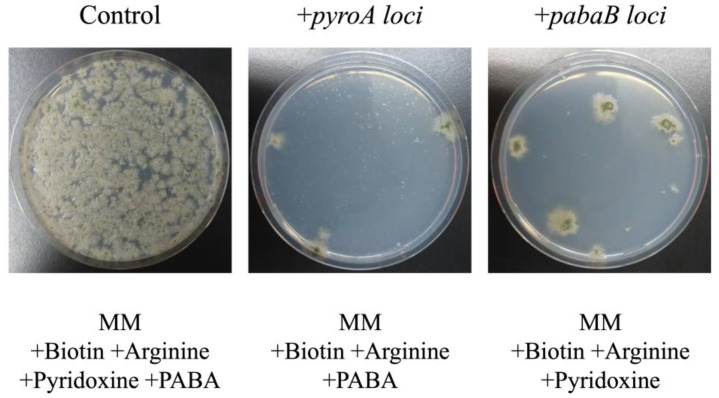
Plates coated with AKU89PPAP transformed with pTOCK1-gAKU89; MM supplemented with biotin, arginine, pyridoxine, and 4-aminobenzoic acid (PABA) (**left** panel) as a positive control; MM supplemented with biotin, arginine, and PABA to select *pyroA* complementation strains (**middle** panel); MM supplemented with biotin, arginine, and pyridoxine to select *pabaB* complementation strains (**right** panel); all plates were incubated at 37 °C for 3 days.

To obtain a triple mutant strain of *pyrG*, *pyroA*, and *pabaB*, AKU89PPAP was constructed by crossing AKU89P (Δ*pyrG*) with the A426 (*yA2*, *pyroA4*, and *pabaB22*) strain. We investigated if AKU89PPAP could be transformed using pTOCK1-gAKU89 to obtain complementary transformants of auxotrophies for pyridoxine and 4-aminobenzoic acid. AKU89PPAP was transformed with 1.5 µg of pTOCK1-gAKU89 and selected on the MM agar medium without uracil and uridine and without pyridoxine or 4-aminobenzoate. In total, 3–6 colonies of pyridoxine- or 4-aminobenzoate-prototrophs were obtained under the conditions that yielded approximately 3.0 × 10^5^ colonies (Figure 2). Thus, pTOCK1-gAKU89 can be used to obtain the desired strain for auxotrophic selection.

### 3.3. Cosmid Rescue Techniques Using In Vitro Packaging

**Figure 3:**
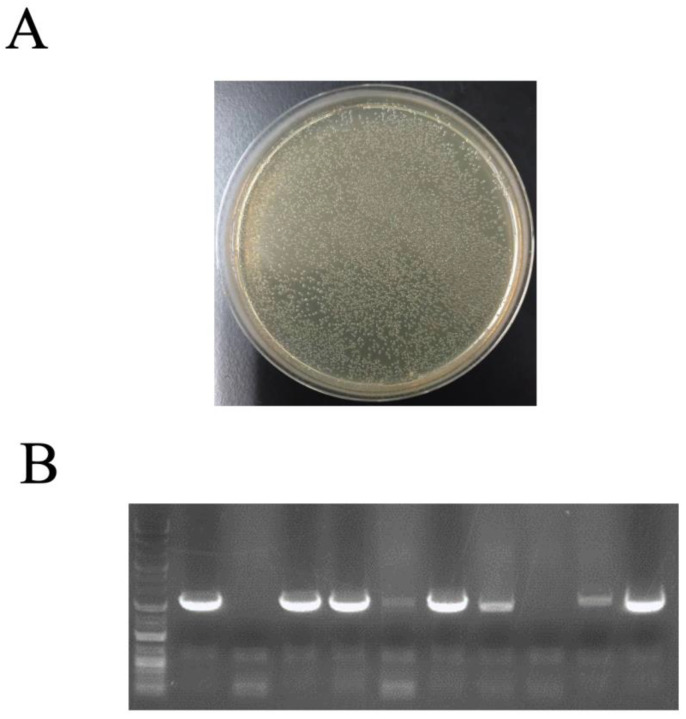
Cosmid rescue technique using in vitro packaging; (**A**) colonies of *Escherichia coli* ED8767 propagated after in vitro packaging with 0.36 µg of the genome of the AKU89PPAP + *pyroA* candidate strain; (**B**) Ten colonies were randomly selected for colony PCR for *pyroA*.

To investigate the rescue efficiency of cosmid libraries, in vitro packaging was performed using genomic DNA extracts from *A. nidulans* transformants. Genomic DNA was extracted from the transformants. In vitro packaging with 0.35 µg of genomic DNA was followed by infection with *E. coli* ED8767. A large number of colonies were obtained, indicating that plasmid rescue is possible even with small amounts of genomic DNA (Figure 3A). Ten colonies were randomly selected for colony PCR using the primer pair pyroA-F–pyroA-R for pyroA. In total, 8/10 colonies had a genomic region containing the *pyroA* locus (Figure 3B), implying that cosmid-based genomic libraries can easily screen and recover clones containing the gene fragment of interest with high efficiency.

### 3.4. Complementation of gfsA with Cosmid Libraries

**Figure 4:**
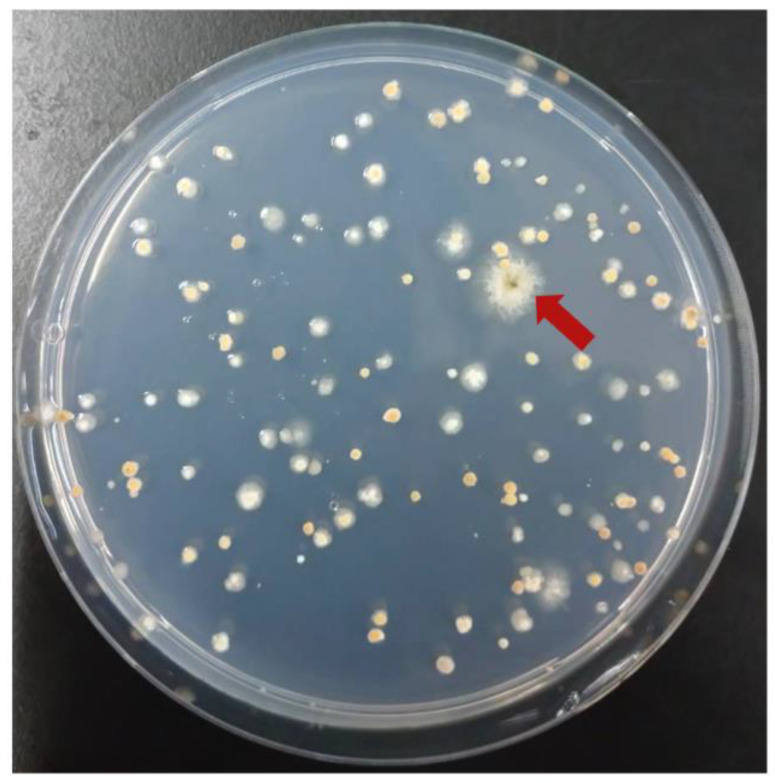
Complementation of the *gfsA* mutant by pTOCK1-gAKU89; Δ*gfsA* Δ*pyrG* was transformed with pTOCK1-gAKU89 and incubated on MM supplemented with biotin at 37 °C for 3 days. Transformants were diluted such that 100 colonies appeared on one plate and were applied to several plates. Red arrows indicate transformants complemented with *gfsA*.

To investigate the possibility of complementing genes that are not subject to selective force, we evaluated whether cosmid clones could be obtained to improve the growth of the *gfsA* mutant [26,31,32]. *A. nidulans* Δ*gfsA* was introduced with 1.5 µg of pTOCK1-gAKU89 and selected on the MM agar medium without uracil and uridine. Although Δ*gfsA* has a significantly smaller colony diameter and barely forms microspores, some colonies emerged with an improved phenotype (Figure 4). The presence of the *gfsA* region on cosmids recovered from strains with improved growth was confirmed by colony PCR. The results confirm that the cosmid library is a powerful genetic analysis tool used for screening in the absence of selective forces, such as nutrient requirement or drug resistance.

### 3.5. Complementation of yA with Cosmid Libraries

**Figure 5:**
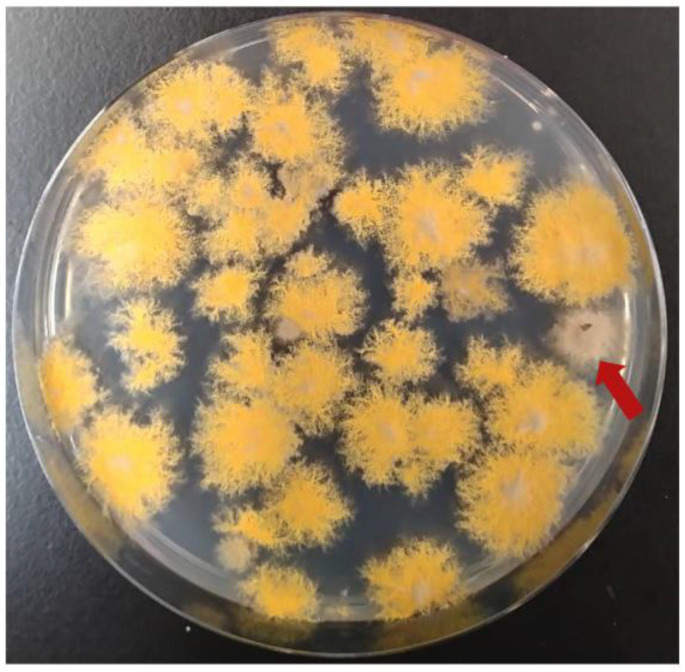
Complementation of the *yA* mutant by pTOCK1-gAKU89; AKU89PY was transformed with pTOCK1-gAKU89 and incubated on MM supplemented with arginine and biotin at 37 °C for 3 days. Transformants were diluted such that 100 colonies appeared on one plate and applied to several plates. Red arrows indicate that the transformants complement *yA*.

To investigate whether transformants with altered secondary metabolite production could be obtained, we examined whether the transformants could be obtained where yellowish conidia of Δ*yA* change to the original greenish conidia. *A. nidulans* AKU89PY was introduced with 1.5 µg of pTOCK1-gAKU89 and selected on the MM agar medium without uracil and uridine. Many transformants (~5000 colonies were examined) formed yellowish conidia, whereas 1 transformant formed conidia showing wild-type coloration (Figure 5). Thus, the cosmid library can be used to screen for transformants with altered secondary metabolic production using colony coloration as an indicator.

### 3.6. Reamplification of Cosmid Libraries

**Figure 6:**
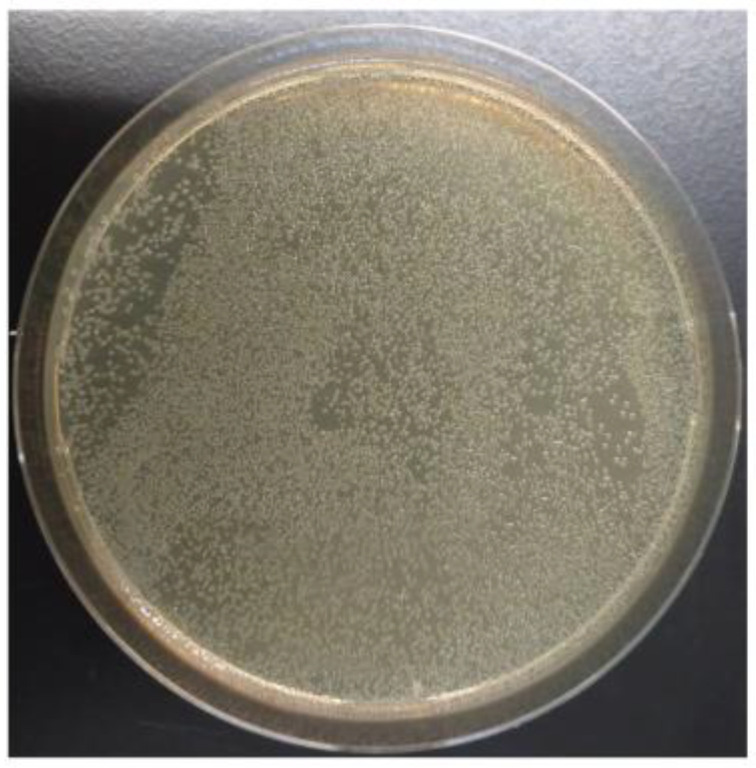
Reamplification of pTOCK1-gAKU89 using in vitro packaging; reconstituted lambda phage was constructed using 0.06 µg of pTOCK1-gAKU89 and propagated through *Escherichia coli* ED8767.

This cosmid library can be easily replicated by culturing *E. coli* containing the cosmid library. Herein, in vitro packaging with cosmid extracts was performed to determine if the extracted cosmid libraries could be repopulated. Reconstituted lambda phages were infected with *E. coli* ED8767 after in vitro packaging with 0.06 µg of cosmid extract. Results showed that a large number of colonies emerged (Figure 6), indicating that the cyclic cosmid pTOCK1-gAKU89 could be reamplified by reconstitution into lambda phage.

## 4. Discussion

**Figure 7:**
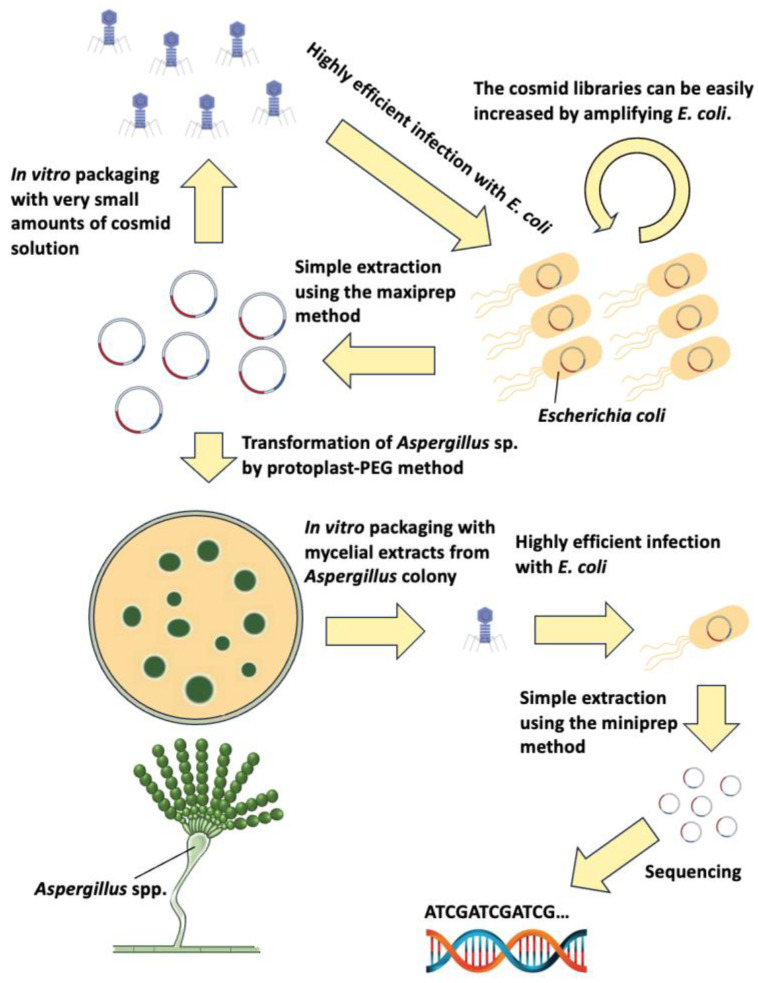
Overall view of the *E. coli*-filamentous fungi shuttle cosmid-based library system.

Herein, we constructed a cosmid vector for *Aspergillus* spp. to prepare tools for efficient genetic analysis. Figure 7 shows the summary of the *E. coli*–filamentous fungi shuttle cosmid-based library system. First, the cosmid library can be amplified by in vitro packaging and infecting *E. coli* several times as necessary, even when a small amount of cosmid solution is available. It can also be easily amplified by culturing the *E. coli* population containing the cosmid library. Large quantities of high-quality cosmid can be easily extracted using the maxiprep method. Cosmids can be introduced into *Aspergillus* spp. with high efficiency and easily transferred back from *Aspergillus* to *E. coli*. Cosmids in *E. coli* can be amplified and sequenced easily; this cosmid is highly versatile and can be used similarly at least for *Aspergillus* spp.

The constructed cosmid vectors were used to generate a genomic library of *A. nidulans*, and their efficiency was demonstrated. The library size of pTOCK1-gAKU89 reached 54,000 clones, indicating that this genomic library has approximately 100% genome coverage. Our cosmid vectors allowed the insertion of 21.1–37.1-kbp-long DNA fragments, achieving highly efficient genome coverage for the first time. A well-recognized genomic library for *A. nidulans* has been constructed based on the plasmid pRG3-AMA1, with an average insert length of approximately 5 kbp [33,34]. This library has been used to identify genes involved in nitrosative stress tolerance in *A. nidulans* [35,36,37]. In addition, several suppressors have been identified that ameliorate the reduced secondary metabolic productivity of Δ*laeA* using a genomic library [38,39]. The analysis of multicopy suppressors may allow the discovery of genetic relationships between genes of known function or novel functions. Recently, the novel transcription factor *socA* has been identified in a genome library as a multicopy suppressor of a strain showing the fully phenotype because of mutations in the O-mannosyltransferase gene *pmtC* [40]. These studies demonstrate that a good genomic library is a powerful tool for identifying the function of novel genes. Notably, genes identified through genomic libraries, unlike those identified through reverse genetics, may be unexpectedly discovered. However, this type of plasmid-based genomic library is difficult to reamplify through *E. coli* retransformation, and library size is drastically reduced each time the library is amplified. Moreover, the *Aspergillus* genome libraries (pRG3-AMA-NotI, pRG3-AMA-niiA, and pRG3-AMA-niaD) were earlier available from the Fungal Genetics.

Stock Center but were not available approximately 20 years ago. Our cosmid-based genomic library can be easily reamplified without reducing the library size with just a small amount of cosmid solution (Figure 6 and Figure 7), addressing these issues. 

The ability of pTOCK1-gAKU89 to complement causal genes using mutant *A. nidulans* strains was tested, suggesting that the desired strain could be obtained with a single transformation (Figure 2, Figure 3, Figure 4 and Figure 5); this can be used to identify new resistance genes—e.g., resistance to drugs—because more colonies can be selected under the selection pressure. In cosmid libraries of *A. niger* constructed by other groups, complementary strains of the transcriptional regulator *prtT* and UDP-galactopyranose mutase (*ugmA*) can be selected [20,41]. Because candidates can be obtained with high efficiency, indicators (e.g., colony coloration) can be used to obtain novel activators of secondary metabolism, which is similar to plasmid-based genomic libraries. A drawback of conventional genomic libraries is that they require high concentrations of genomic DNA for plasmid rescue. Our cosmid vectors enable highly efficient plasmid rescue even with small amounts of genomic DNA by in vitro packaging, making it easy to recover target clones. The results of colony PCR of *E. coli* clones obtained by plasmid rescue revealed that approximately 80% of the clones contained the loci of interest (Figure 3B). By contrast, 20% of the cosmids were obtained without the *pyroA* locus. Plasmid rescue can yield numerous empty vectors, suggesting that the fugal transformant has multiple plasmids introduced. Plasmid rescue using electroporation or the use of chemically competent cells has low transformation efficiency and may pick *E. coli* clones with empty vectors. During transduction by in vitro packaging, the infection efficiency is so high that the ease of recovering the cosmid of interest, even with low concentrations of genomic DNA, is an advantage for genetic analysis. Herein, several pTOCK1-gAKU89 clones were obtained with multiple fragments of another chromosomal origin inserted. Although this is an undesirable quality, nanopore sequencers can be used to analyze sequences of plasmids as large as 50–100 kbp at low cost. Therefore, pTOCK1 may be useful as a powerful tool for genetic analysis in filamentous fungi. Because sequences of up to 40 kbp can be cloned in cosmids, entire lengths of secondary metabolic gene clusters can be cloned [42,43,44,45].

In conclusion, our studies of cosmid-based genomic libraries of *A. nidulans* revealed that genetic analysis can be conducted conveniently in filamentous fungi. Cosmid-based genomic libraries can be used to identify the causative genes of mutant strains in filamentous fungi. The revival of genetic research on filamentous fungi will advance bioengineering and fermentation industries to develop antifungal drugs against pathogenic fungi. 

## Figures and Tables

**Table 1:** Strains used in this study.

Strains	Genotype	Source
*Aspergillus nidulans*		
A26	*biA1*	obtained from FGSC
A426	*yA2*, *pabaB22*, *pyroA4*	obtained from FGSC
AKU89	*biA1*, *argB2*, *nkuB*::*aurA*^+^	Goto et al., 2009 [25]
AKU89P	*biA1*, *argB2*, *nkuB*::*aurA*^+^, Δ*pyrG*	This study
AKU89PPAP	*biA1*, *argB2*, *nkuB*::*aurA*^+^, Δ*pyrG*, *pabaB22*, *pyroA4*	This study
Δ*gfsA*	*biA1*, *argB2*, *nkuB*::*aurA*^+^, *gfsA*::*argB*	Komachi et al., 2013 [26]
Δ*gfsA* Δ*pyrG*	*biA1*, *argB2*, *nkuB*::*aurA*^+^, Δ*pyrG*, *gfsA*::*argB*	This study
AKU89PY	*biA1*, *argB2*, *nkuB*::*aurA*^+^, Δ*pyrG yA*::*nat1*	This study

**Table 2:** Primers used in this study.

SuperCos1-PmaCI-F2	AAAACACGTGTACGTCTGCTTTTTGTTGACTTCC	This study
SuperCos1-PmaCI-R2	AAAACACGTGCACGTCTGAAGCTAGCTTCGA	This study
AoPyrG-Scos1-infusion(AatII)-F	GAAAAGTGCCACCTGTATGGATCTCAGAACAATATACC	This study
AoPyrG-Scos1-infusion(AatII)-R	ATAATGGTTTCTTAGTGTACGATAGTGACCGACTG	This study
pyrG-del-1	AGCTTAAAGAAAACGCGCAGC	This study
pyrG-del-2	ACTAGAGCCGTCAGTGAGGCGATGGCGGTTCTCCAATGAT	This study
pyrG-del-3	ATCATTGGAGAACCGCCATCGCCTCACTGACGGCTCTAGT	This study
pyrG-del-4	GATGGTGAGGATAGGAATTGCC	This study
pyrG-FC	GTATCTTTCCCCCTTCAACGC	This study
pyrG-RC	GATCAAGGAAAGGGGCGAGG	This study
AN8677-7	CGCGTAGATCACACAGGGTC	Komachi et al., 2013 [26]
AN8677-8	GCATTGTTCGATCGCTCGTC	Komachi et al., 2013 [26]
AN8677-FC	GCTCGAATCTTCCGTATGCC	This study
AN8677-RC	GTCCCGCCAATAATTTTAGCCATA	This study
pyroA-F	AGTAGAGGCAATATAGTCCCTGCG	This study
pyroA-R	CAGATGTAAATGTCAAAAGGTCCGTC	This study
nat1-F	ATGGCCACCCTCGACGACACGGC	This study
nat1-R	TCAGGGGCAGGGCATGCTCATG	This study
AnGpdA-P-TrpC-T-F(nat1)	ATGCCCTGCCCCTGAGGATCCACTTAACGTTACTGAAATC	This study
AnGpdA-P-TrpC-T-R(nat1)	GTCGAGGGTGGCCATGGTGATGTCTGCTCAAGCGG	This study
yA-1	CGGGCTGCAGGAATTCCCCGACAAGTTTCTCAGTGG	This study
yA-2	AGAGTCGACCCCTCGGCTGGATCCCGGAGGAATCA	This study
yA-3	CCCATCGATGGGGTAAAATGGGAGGAATGGCGCTG	This study
yA-4	CCCATCGATGGGGTAATTGCCCTCCCTGGCGTATA	This study
yA-FC	GGACCTGACAATTCTCGACGT	This study
yA-RC	GCCGGTCAAAACTGCATCGG	This study

## Data Availability

All the data appear in the manuscript figures and tables.
